# Comparing life history traits and tolerance to changing environments of two oyster species (*Ostrea edulis* and *Crassostrea gigas*) through Dynamic Energy Budget theory

**DOI:** 10.1093/conphys/coac034

**Published:** 2022-07-08

**Authors:** Brecht Stechele, Marie Maar, Jeroen Wijsman, Dimitry Van der Zande, Steven Degraer, Peter Bossier, Nancy Nevejan

**Affiliations:** Laboratory of Aquaculture & Artemia Reference Center, Ghent University, Coupure Links 653, B-9000 Gent, Belgium; Department of Bioscience, Applied Marine Ecology and Modelling, Aarhus University, Frederiksborgvej 399, 4000 Roskilde, Denmark; Wageningen University and Research, Wageningen Marine Research, PO Box 77, Korringaweg 7, 4400AB, Yerseke, The Netherlands; Operational Directorate Natural Environment, Royal Belgian Institute of Natural Sciences, 29 Vautierstraat, 1000 Brussel, Belgium; Operational Directorate Natural Environment, Royal Belgian Institute of Natural Sciences, 29 Vautierstraat, 1000 Brussel, Belgium; Laboratory of Aquaculture & Artemia Reference Center, Ghent University, Coupure Links 653, B-9000 Gent, Belgium; Laboratory of Aquaculture & Artemia Reference Center, Ghent University, Coupure Links 653, B-9000 Gent, Belgium

**Keywords:** climate change, Dynamic Energy Budget, European flat oyster, life history traits, Pacific cupped oyster

## Abstract

To predict the response of the European flat oyster (*Ostrea edulis*) and Pacific cupped oyster (*Crassostrea gigas/Magallana gigas*) populations to environmental changes, it is key to understand their life history traits. The Dynamic Energy Budget (DEB) theory is a mechanistic framework that enables the quantification of the bioenergetics of development, growth and reproduction from fertilization to death across different life stages. This study estimates the DEB parameters for the European flat oyster, based on a comprehensive dataset, while DEB parameters for the Pacific cupped oyster were extracted from the literature. The DEB parameters for both species were validated using growth rates from laboratory experiments at several constant temperatures and food levels as well as with collected aquaculture data from the Limfjorden, Denmark, and the German Bight. DEB parameters and the Arrhenius temperature parameters were compared to get insight in the life history traits of both species. It is expected that increasing water temperatures due to climate change will be beneficial for both species. Lower assimilation rates and high energy allocation to soma explain *O. edulis’* slow growth and low reproductive output. *Crassostrea gigas’* high assimilation rate, low investment in soma and extremely low reserve mobility explains the species’ fast growth, high tolerance to starvation and high reproductive output. Hence, the reproductive strategies of both species are considerably different. Flat oysters are especially susceptible to unfavourable environmental conditions during the brooding period, while Pacific oysters’ large investment in reproduction make it well adapted to highly diverse environments. Based on the life history traits, aquaculture and restoration of *O. edulis* should be executed in environments with suitable and stable conditions.

## Teaser

European flat oysters (*Ostrea edulis*) and Pacific cupped oyster (*Crassostrea gigas*) have distinct differences in Dynamic Energy Budget parameters. These parameters explain their history traits and life strategies when exposed to changing environments in Northern Europe.

## HIGHLIGHTS

1. The DEB parameters for *O. edulis* are estimated and validated.
2. The temperature response of *O. edulis* and *C. gigas* is estimated and validated.
3. Increasing temperatures due to climate change will increase the suitability of European waters for both *C. gigas* and *O. edulis*.
4. *Ostrea edulis* prefers stable environmental conditions, while *C. gigas* can cope better with dynamic systems and starvation.
5. Concentrating *O. edulis* restoration and aquaculture projects to stable environments will increase success.

## Introduction

The European flat oyster (*Ostrea edulis*) is indigenous to Europe and has been part of the human diet since prehistoric times (Kristensen, 1997; Goulletquer, 2004; Gercken and Schmidt, 2014). Oyster spat (attached juveniles) has historically been collected from rocks for grow-out in ponds (Günther, 1897), and oyster reefs supported a thriving fishery (Eyton and van Voorst, 1858; Dean, 1893). Overfishing, the introduction of diseases (e.g. *Bonamia spp.*) and several cold winters devastated wild oyster stocks and aquaculture practices since the 18th century (Airoldi and Beck, 2007; Thurstan *et al.,* 2013; Gercken and Schmidt, 2014). The species is listed as a ‘Threatened and Declining species’ by the Convention for the Protection of the Marine environment of the North-East Atlantic (OSPAR) convention for the Protection of the Marine Environment of the North-East Atlantic (Haelters and Kerckhof, 2009). At present, small relic populations remain present in European waters (Launey *et al.,* 2002; De Mesel *et al.,* 2018; Nielsen and Petersen, 2019; Thorngren *et al.,* 2019). Nevertheless, historical, cultural, economic and ecological value of the species has recently kick-started several aquaculture, conservation and restoration projects (see Native Oyster Restoration Alliance, https://noraeurope.eu/).

After the collapse of the flat oyster culture in Europe, the Pacific oyster (*Crassostrea gigas*) was introduced in the 1960s to meet the high demand for oysters (Shatkin, 1997; Drinkwaard, 1998; Reise *et al.,* 1998; Smaal *et al.,* 2009). Although European waters were initially assumed too cold for reproduction, Pacific oysters began spreading into the natural environment and are now competing with flat oysters because they have overlapping habitat and food preferences and similar tolerance to temperature and salinity (Drinkwaard, 1998; Wehrmann *et al.,* 2000; Wrange *et al.,* 2010; Nielsen *et al.,* 2017). Suitable growing conditions boosted production and in 2018, the Pacific cupped oyster represented 95% of the EU’s oyster aquaculture with 100 000 tonnes produced per year (Wijsman *et al.,* 2019), while flat oyster cultivation became marginal (STECF-18-19). Although, currently, flat oyster aquaculture is limited, (the sector valued USD 12441 in 2017 and represented 1703 tons of production according to Food and Agriculture Organization (FAO) (FAO, 2020)), flat oysters are generally priced around €6/kg where Pacific oyster are around €4/kg (STECF-18-19). *Ostrea edulis* products occupy a niche and are considered a luxury sea food item, destined for specialized consumers (Towers, 2010).

Both aquaculture and restoration efforts need comprehensive understanding of oysters’ environmental requirements and life strategies. Environmental changes due to climate change, such as warmer sea water, heat waves or altered river runoff patterns, can affect oyster growth rate, reproduction and mortality, and hence impact the success of aquaculture and restoration efforts or change the balance in competition between both species. Comparing life history traits is key in understanding how both species react to changing environments (Cole *et al.,* 2016; Gray *et al.,* 2019). One trait that plays an important role is the reproductive strategy. In comparison with the Pacific oyster, the European flat oysters are typified by a relatively small quantity of offspring (range of hundred thousands) corresponding to an increased parental investment through brooding. The Pacific oyster, on the other hand, is adapted to spatially heterogeneous and/or temporally varying environments by the ability to produce large batches of offspring (range of millions) (Levins, 1963) and reproduces through broadcast spawning. The Dynamic Energy Budget (DEB) theory provides a quantitative framework to describe the aspects of a species metabolism and its life history traits (Kooijman, 2010). Ecophysiological characteristics, such as the differentiation between r- or K-strategists, can be explained through the DEB theory, and comparing DEB parameters for *O. edulis* and *C. gigas* gives quantitative insight into their response to changing environments.

This study provides the DEB parameters for the European flat oyster based on a comprehensive dataset, whereas the DEB parameters for Pacific oyster are obtained from previous studies (Pouvreau *et al.,* 2006; Bourlès *et al.,* 2009; Emmery and Kooijman, 2015). The aim of the study is to compare life history traits, competition and tolerance to changing environments of both oyster species based on DEB theory.

## Material and methods

### DEB model

The individual bioenergetic models used in this study is based on the DEB theory established by Kooijman (2010). The DEB theory is a generic theory that is applicable to all organisms through species-specific DEB parameters (Kooijman, 2010; Lika *et al.,* 2011). DEB describes the energy dynamics of an individual organism based on four state variables: reserve energy, $E$ (J); body structural volume, $V$ (*cm^3^*); the reproduction buffer, ${E}_R$ (*J*); and the cumulative investment into development, called maturity, ${E}_H$ (*J*) in DEB terminology. Maturity levels are used to differentiate between life stages and start with a value of 0 J at fertilization. Before birth (${E}_H<{E}_H^b$), no feeding occurs, and individuals will use reserve energy to develop and grow. After birth, the individual will start feeding, and food availability relates to the ingestion (${\dot{p}}_X$) and assimilation of energy (${\dot{p}}_A$) in the general reserve. From the reserve, energy is mobilized $({\dot{p}}_C)$. A fraction $(\kappa )$ of the mobilized energy, will be used for somatic growth $({\dot{p}}_G)$ and organism functional maintenance $({\dot{p}}_S)$, while the remaining fraction $(1-\kappa )$ of the mobilized energy will be allocated to development $({\dot{p}}_R)$ if the individual has not yet reached puberty (${E}_H<{E}_H^p$) or the reproduction buffer if the individual has fully matured (${E}_H={E}_H^p$). Development goes with a maintenance cost, which is called maturation maintenance $({\dot{p}}_J)$.

The standard DEB model describes the change in state variables over the course of three main life stages, i.e. embryo (${E}_H<{E}_H^b$), juvenile (${E}_H^b<{E}_H<{E}_H^p$) and adult (${E}_H^p={E}_H$). The DEB model for flat oyster parameterized in this study also integrates metabolic changes during the pelagic larval phase and the metamorphosis, and is therefore classified as the ‘asj’ typified model with metabolic acceleration (a) between settlement (s) and the juvenile phase (j); ${E}_H^s<{E}_H<{E}_H^j$, with ${E}_H^s$ and ${E}_H^j$ the maturity at settlement and at the end of metamorphosis (Kooijman, 2010, 2014). During metamorphosis, growth is exponential at constant food (Marques *et al*., 2019). This type of acceleration is commonly used to describe the metabolism of species such as the black-lipped pearl oyster (*Pinctada margaritifera*) (Sangare, 2019; Sangare *et al.,* 2019, 2020) and the Pacific oyster (Emmery and Kooijman, 2015). When focusing only on the juvenile/adult phase, an asj-model can easily be simplified to a standard model by multiplying parameter $\{{p}_{Am}\}$ and $\dot{v}$ with the acceleration factor. A detailed model description is added as supplementary material.

#### Parameter estimation

**Figure 1:**
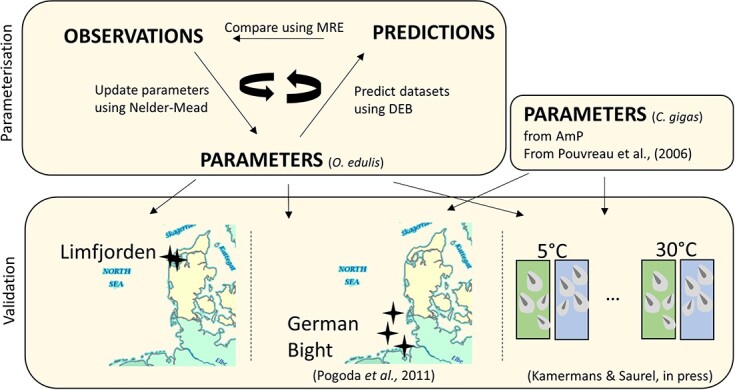
Methodology overview: DEB parameters of *O. edulis* are estimated using the covariate method based on the minimalization of the loss function. DEB parameters of *C. gigas* were retrieved from the literature (AmP, 2021). Parameters of both oysters were validated using aquaculture growth data from the Limfjorden, Denmark, and the German Bight (Pogoda *et al.,* 2011) and experimental growth data at different food levels and temperatures (Kamermans and Saurel, 2022).

The DEBtool (2021) software packages was used for parametrizing the model (https://add-my-pet.github.io/DEBportal/docs/AmPestimation.html). The Add my Pet routine (AmP, 2021) included in the DEBtool allows for simultaneous estimation of the species-specific primary DEB parameters from empirical data (Fig. 1). DEB parameters were parameterized using various zero and univariate datasets retrieved from published literature studies.

The four standard DEB estimation files are used for the prediction of the DEB parameters. The ‘mydata file’ contains all datasets used for parametrization. The ‘pars_init file’ holds the initial parameters, from which optimization starts. In the ‘predict file’, the DEB formulation calculates the life history traits of the organism based on the proposed primary parameters. These predictions are compared with the datasets provided, using a loss function. Minimization of the loss function happens through the co-variation method (Lika *et al.,* 2011), which is an iterative optimization routine based on the Nelder–Mead simplex algorithm. Optimization is based on the minimization of the weighted squared residuals between model and observations (relative error, RE). The mean relative error (MRE) was estimated by

**Figure 2:**
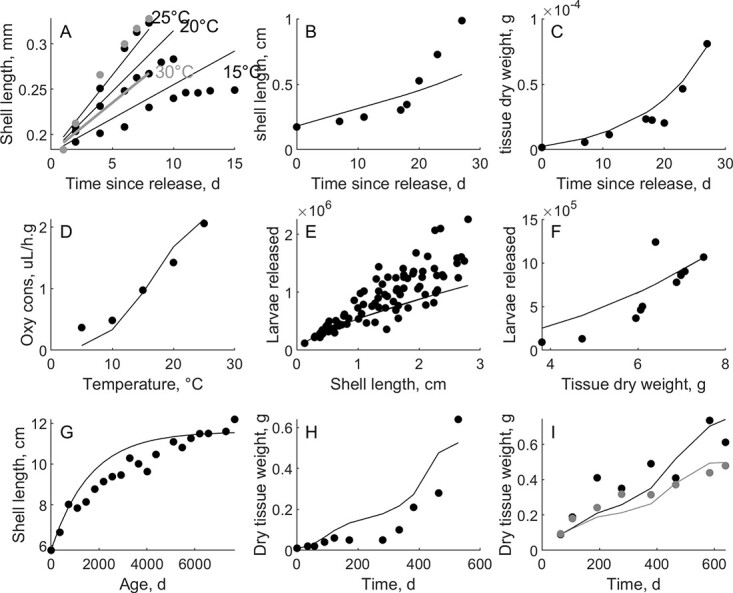
Flat oyster observations (dots) from published literature versus predictions through DEB (lines). The data cover various life stages and environments as well as several aspects of metabolism. (**A**) Larval growth at different temperatures (Robert *et al.,* 2017). (**B**,**C**) Larval and post-larval length and dry tissue weight measured starting from release (Labarta *et al.,* 1999). (**D**) Oxygen consumption at different temperatures (Hutchinson and Hawkins, 1992). (**E**,**F**) Reproductive output related to weight and length (Cole, 1941; Walne, 1964). (**G**) Shell length versus age measured during a population analysis of a wild stock based on acetate peels (Saurel and Richardson, 2003). (**H**) Aquaculture growth in Galway (Wilson, 1987). (**I**) Aquaculture growth at two different locations in Arcachon, France, of Bonamia-infected oysters (Robert *et al.,* 1991).

\begin{align*} MRE=\sqrt{\frac{\sum_{i=1}^n{\sum}_{j=1}^{m_i}{\beta}_{ij}\ {\left(\frac{Y_{ij}-{\hat{Y}}_{ij}}{Y_{ij}}\right)}^2}{\sum_{i=1}^n{\sum}_{j=1}^{m_i}{\beta}_{ij}}}, \end{align*}

where ${m}_i$ is the number of datapoints in dataset $i$ (and $n$ the number of datasets) and each zero-variate data value is considered to be one dataset; and${Y}_{ij}$, ${\hat{Y}}_{ij}$ and ${\beta}_{ij}$ are the observations, model predictions and weight coefficient, respectively, corresponding to dataset $i$ and datapoint $j$. Datasets (zero variate and univariate) for estimation of the DEB parameters were collected from published literature about flat oysters (Table 1; Fig. 2).

Although the MRE gives the match between available data and estimations of the model, it is still of main importance to judge the realism of parameter values and estimations. Large variations in parameters can often result in small differences in the goodness of fit (Lika *et al.,* 2011).

**Table 1:** Observations (data) versus predictions (prd) of flat oyster characteristics at important life-history events.

Description	Symbol	Data	Unit	prd	RE	Reference
Life span	am	1.28E+04	d	1.19E+04	0.07	Saurel and Richardson, 2003
Age at birth	ab	10	d	5.2	0.48	Boudry *et al.,* 2002
Age at release	ar	10	d	5.8	0.42	Boudry *et al.,* 2002
Length at birth	Lb	0.01	cm	0.0190	0.94	Buroker, 1985
Length at release	Lr	0.0183	cm	0.0200	0.10	Robert *et al.,* 2017
Length at eyespot	Ls	0.03	cm	0.0387	0.29	Rodriguez-Perez *et al.,* 2019
Initial wet weight	Wd0	3.89E-07	g	3.57E-07	0.08	Walne, 1964
Weight at release	Wdr	2.04E-07	g	3.42E-07	0.68	Labarta *et al.,* 1999
Weight at settlement	Wds	2.81E-06	g	2.35E-06	0.16	Labarta *et al.,* 1999
Wet weight at puberty	Wwp	0.19	g	0.20	0.05	Cole, 1941
Ultimate dry weight	Wdi	15.8	g	16.2	0.03	Saurel *et al.,* 2003
Maximum clearance rate	CR_max	12.6	L/h/g	12.6	0.003	Nielsen *et al.* 2017
Ultimate reproduction rate	Ri	2.50E+06	#	3.57E+06	0.43	Walne, 1964

#### Parameter validation

After model parameterization, *O. edulis* DEB parameters were validated using different datasets (Fig. 1). Two growth curves were collected in the Limfjorden, Denmark, during aquaculture trails, and three growth curves were extracted from published grow-out experiments in the German Bight (Pogoda *et al.,* 2011). In addition, experimental growth data collected at different constant food level and temperatures were used to optimize the Arrhenius temperature parameters and to validate the DEB primary parameters (Kamermans and Saurel, 2022). The DEB parameters for *C. gigas*, which have been published in the literature (AmP, 2021), were validated using grow-out experiments in the German Bight (Pogoda *et al.,* 2011) and experimentally collected growth data at constant food and temperature (Kamermans and Saurel, 2022). Food availability was estimated for all validation datasets by using the average chlorophyll *a* (chl) concentration as the food proxy.

All simulations were done using MATLAB version R2019b.

### Validation datasets

#### *O. edulis* growth in the Limfjorden

Validation data was collected from two sites in the Danish Limfjorden, Lemvig Havn and Gjellergraven, by the OysterBoat company. The Lemvig Havn (56.554 N, 8.308E) site is a harbour area, used for culturing of spat (>10 mm) until they reach a size of 30 mm. The spat was placed in one layer on the bottom of a basket (suspended at a depth of 1 m) and around 200 individuals were weighted 32 times from 10 April to 3 December 2014 (Fig. 4). The more open-water station Gjellergraven (56.594 N, 8.316E) is a grow-out area for oysters (>30 mm). The oysters are typically harvested after 3.5 years of culturing. The oysters are placed in net bags (1.2 × 1.2 m) deployed 0.3 m above the bottom (water depth, 5.5 m). For the present study, total wet weight and shell length of an average of 130 oysters was measured seven times between 20 February 2008 and 17 November 2009. Water temperature (Gjellergraven) and salinity (both stations) were obtained from a hydrodynamic model (Pastor *et al*. 2021). In Lemvig Havn, water temperature was measured by a temperature logger (www.orumjensen.dk), while some periods with missing data was substituted by data from the Danish Meteorological institute (stn no. 24032). The average chl concentration at the Lemvig Havn and Gjellergraven site were 6.34 and 2.87 μg chl a L^−1^, respectively, obtained from model results (Maar *et al*., unpublished data). The dry tissue to total weight ratio used by OysterBoat was 0.015.

The initial state variables for the validation step were estimated by recalculating weights to structural length, assuming fit animals ($e=0.8$) with an empty reproduction buffer (${E}_R=0$) and a maximum maturity level (${E}_H={E}_H^p$). During spawning events, a fraction of the reproduction buffer (${\kappa}_R$) is emptied. Reproduction of *O. edulis* was simulated considering a brooding period at which the larvae grow internally until release (${E}_H^r$), food intake by the brooding individual was limited during the brooding period with a factor 0.8, based on Chaparro *et al.* (2001) and Araya *et al.* (2012).

#### *O. edulis* and *C. gigas* growth at constant food and temperature

Kamermans and Saurel (2022) measured growth of *C. gigas* and *O. edulis* under laboratory conditions at different temperatures (3, 8, 15, 20, 25 and 30°C) and two food regimes (2 and 10 μg chl a L^−1^) for 6 weeks. For the experiments, oysters were selected from the same size class; *C. gigas* spat had an average shell length of 21.9 ± 4.5 mm and an average wet weight of 1.13 ± 0.61 g, while *O. edulis* spat had an average shell length of 24.0 ± 4.2 mm and an average wet weight of 0.87 ± 0.44 g. The 12 treatments were each performed in triplicate. One replicate contained 10 individually marked oysters placed in 30 l tanks. Food consisted of two algae species (50% *Skeletonema cosatum* and 50% *Isochrysis galbana V/V*). At the beginning and the end of the experiment, the shell length of the individual oyster was measured with a digital calliper and the total wet weight was measured with a balance. At the end of the experiment, flesh dry weight was measured after drying at 70°C to constant weight. For *C. gigas* the DW/WW-ratio was 0.018 (*n* = 62), and for *O. edulis* the DW/WW-ratio was 0.015 (*n* = 60). Individual growth rates (g DW d^−1^) of the oysters were calculated by the changes in DW over time ($\frac{\Delta DW}{\Delta t}=\frac{DW_{end}-{DW}_{start}}{time}$).

#### *O. edulis* and *C. gigas* growth in the German Bight

Pogoda *et al.* (2011) collected flat and Pacific oyster growth data in the German Bight during the spring, summer and autumn of 2004. At two offshore locations, in the Butendiek wind farm (54.985 N, 7.907E) and near the island of Helgoland (54.190 N, 7.883E), and one inshore location called Wurster Arm (53.678 N, 8.408E) small oyster were deployed in lantern nets. Juvenile oysters (10–20 mm) were stocked in spring and sampled with divers. The average chl concentration at the test sites were 3.4, 3.3 and 11.3 μg chl a L^−1^ (obtained from remote sensing products).

Daily average sea surface temperature estimates were extracted at the test sites in the German Bight from the ‘Global Ocean OSTIA Sea Surface Temperature and Sea Ice Reprocessed’ product provided by Copernicus Marine (Product identifier: SST_GLO_SST_L4_REP_OBSERVATIONS_010_011, 2011). The coherent satellite-based chl products used as forcing data for the German Bight datasets were generated based on data from different ocean colour sensors (i.e. SeaWiFS, MODIS, MERIS, VIIRS, Sentinel-3) following the approach of Van der Zande *et al.* (2019) and Lavigne *et al.* (2021). The daily chl products were generated for 2004. The chl datasets were reconstructed using DINEOF (Data Interpolating Empirical Orthogonal Functions, Alvera-Azcárate *et al.,* 2005). DINEOF calculates the expected value for the missing data based on the spatio-temporal information contained in the dataset.

## Results

### Parameter estimation

The DEB parameters for the *O. edulis* model were estimated using 26 data entries (13 zero-variate datasets and 13 univariate datasets) that span all life stages (larvae, post-larvae, juveniles and adults), various metabolic traits (growth, oxygen consumption, clearance rate and reproduction) and environments (aquaculture growth in France and Ireland; growth in the wild populations in the UK). The parametrization resulted in an average match between observations and simulations quantified by

\begin{align*} MRE=0.245. \end{align*}

Zero-variate data predictions (Table 1) were acceptable ($MRE<0.2$), apart from predictions for the first important life history events (birth, release and settlement). The age at release was underestimated ($RE=0.41$), while the length and dry tissue weight at release were overestimated ($RE=0.10$ and $0.67$, respectively). The model predictions indicate that the start of feeding is closely linked to the moment of release. The shell length at settlement ($MRE=0.29$) was overestimated but the tissue dry weight at settlement was underestimated ($MRE=0.16$). Predicted life history traits at puberty or at ultimate age had an acceptable fitting in comparison with the observations. The ultimate reproduction rate was overestimated ($MRE=0.42$), but the only adequate reproduction data at larger oyster sizes was given by Walne (1964) and accounted for animals with a size considerably smaller than the ultimate size.

Although RE values of the zero-variate data predictions of larval phases were high, growth data pertaining to the free-swimming larval stage (Robert *et al.,* 2017) was predicted well (Fig. 2A; $MRE=0.08$). Linear growth was predicted between release and settlement at an estimated food availability of $f=0.6$, indicating average feeding conditions that is expected in a hatchery, where larvae are fed a diet with limited diversity. Larval growth at high temperatures (30°C) was underestimated, indicating the need for validation of the temperature parameters for the larval stages. Growth data before, during and after settlement was collected by Labarta *et al.* (1999). The predictions of growth during these important life stages (Fig. 2B,C) were acceptable. Dry weight predictions corresponded to organic dry weight measurements of the larvae and post larvae ($MRE=0.22$), both before and during acceleration. The decrease in dry weight between Days 15 and 20 was due to the moment of settlement, restructuring of organs such as the loss of the velum and a consequently reduction in filtration rate during this period. After settlement, shell length increases faster than predicted. The sudden change in shell length increase could not be grasped by the model ($MRE=0.29$). DEB assumes isomorphy over the whole life cycle, but shell length data show a clear deviation from this assumption. The oxygen consumption (Fig. 2D) was predicted well with underestimations at lower temperatures and overestimations at higher temperatures ($MRE=0.15$). Predictions of reproduction data (Fig. 2E, F,) collected by Cole (1941) and Walne (1964) were accurate, but the high scatter on the data resulted in a low precision of the model ($MRE=0.29$ and $0.27$). Age-size relation observed in wild stocks (measurement of umbo growth lines), collected by Saurel *et al.* (2003), could be reproduced well by the model ($f=0.9; MRE=0.06$). Simulations of aquaculture growth in Ireland (Wilson, 1987) resulted in a correct trend, but growth during the winter was overestimated when applying constant food ($f=0.9; MRE=0.52$). Oyster growth in the bay of Arcachon (Robert *et al.,* 1991) was simulated at two locations, and predictions fitted the data when using low constant food levels ($f=0.44/0.52; MRE=0.28/0.15$) and variable temperature. Slow growth was appointed to the intertidal nature of cultivation and a *Bonamia* infection in the oysters.

### Validation

Several datasets were included in the validation of the DEB parameters for flat and Pacific oysters. The half-saturation constant was optimized to link the average chl concentration to food availability and was estimated to be ${K}_X=1.84\ \mu g\ \textrm{chl}\ {L}^{-1}$for the flat oyster and ${K}_X=3.29\ \mu g\ \textrm{chl}\ {L}^{-1}$for Pacific oyster (based on all validation datasets). The validation resulted in a goodness of fit of $MRE=0.12$ for flat oyster and $MRE=0.21$ for Pacific oyster.

**Figure 3:**
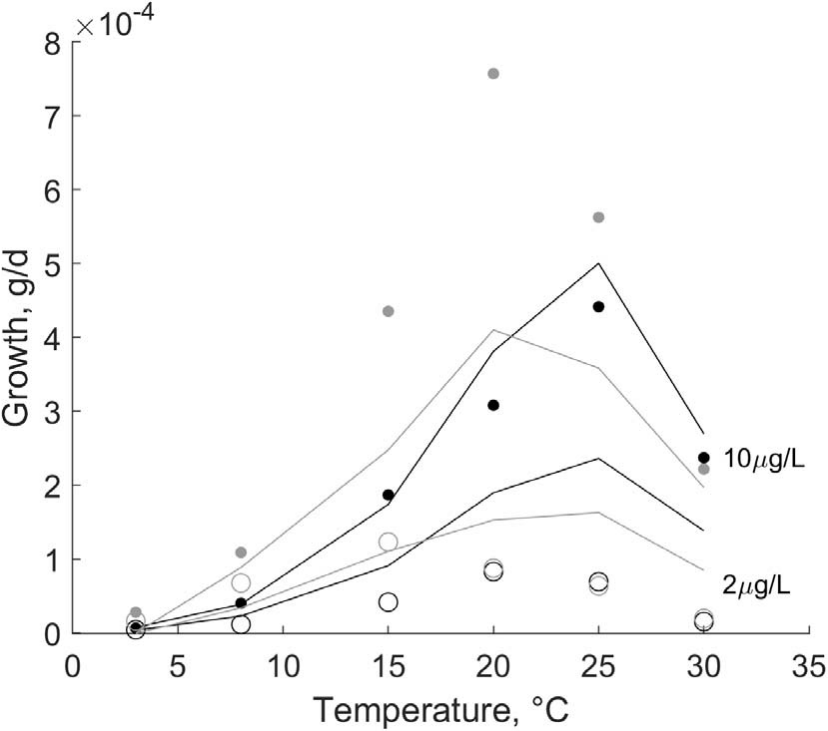
Observed (dots) and predicted (lines) growth rate of *O. edulis* (black) and *C. gigas* (grey) at temperatures ranging from 3°C to 30°C. Oysters were fed a high-algae (10 μg L-1) diet (*O. edulis*, *f* = 0.84; *C. gigas*, *f* = 0.75) and a low-algae (2 μg L-1) diet (*O. edulis*, *f* = 0.52; *C. gigas*, *f* = 34).

The estimations of growth at constant food levels of 2 μg chl L^-1^ and 10 μg chl L^-1^ and temperatures ranging from 3°C to 30°C (Kamermans and Saurel, 2022) were simulated for flat and Pacific oyster (Fig. 3). Flat oyster growth ceases at a temperature <8°C and the optimal growth range stretches up to temperatures of 30°C. Growth rates are overestimated at 2 μg chl L^−1^ ($f=0.52; MRE=0.18$) and match the data well at 10 μg chl L^−1^ ($f=0.84; MRE=0.04$). The effect of temperature on growth of Pacific oysters was represented well, but growth was overestimated at optimal temperatures and low food conditions ($f=0.38; MRE=0.05$) and underestimated at optimal temperatures and high food conditions ($f=0.75; MRE=0.11$). Pacific oysters showed growth, although small, at temperatures <8°C.

**Figure 4:**
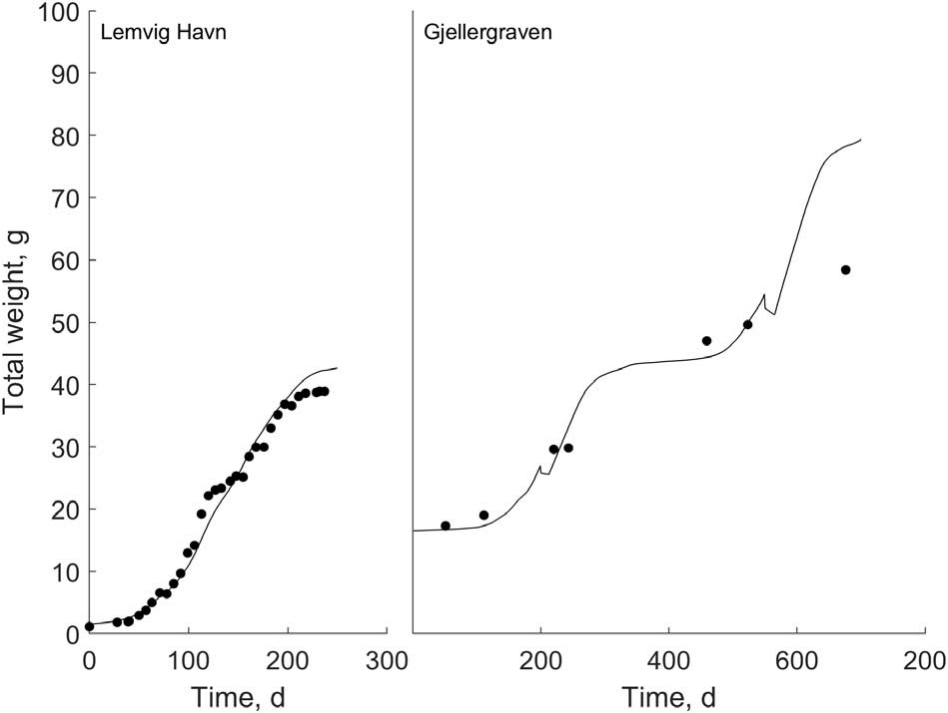
Aquaculture growth estimates at two locations in the Limfjorden, Denmark, at variable temperature and constant food. Data were collected during 2014 in Lemvig Havn (*f* = 0.79) and from 2008 to 2010 in Gjellergraven (*f* = 0.60).

In the Limfjorden, Denmark, both at the Lemvig Havn and at the Gjellergraven site, flat oyster growth data was collected by measuring the total wet weight of the oysters (Fig. 4). Conditions at the Lemvig Havn were ideal and very fast growth was seen ($f=0.79$). Growth rates shortly decreased during the reproductive season (between Days 100 and 150), although no reproduction was witnessed by the farmer. The model to data fitting resulted in a good fit quantified by an $MRE=0.06$. The Gjellergraven site is mainly used for further grow-out of juveniles priorly cultivated at Lemvig Havn. Growth was monitored less frequently compared with in the Lemvig Havn test site. chl concentrations and the corresponding food availability was slightly lower compared with Lemvig Havn ($f=0.60)$. Simulations resulted in an overestimation of growth during the third growth season ($MRE=0.11$). However, the oyster farmer reported that settling of ascidians on top of the basket in November 2009 hindered an efficient food flux and probably limited growth of the oysters. The model estimates showed a decrease in weight during brooding and spawning.

**Figure 5:**
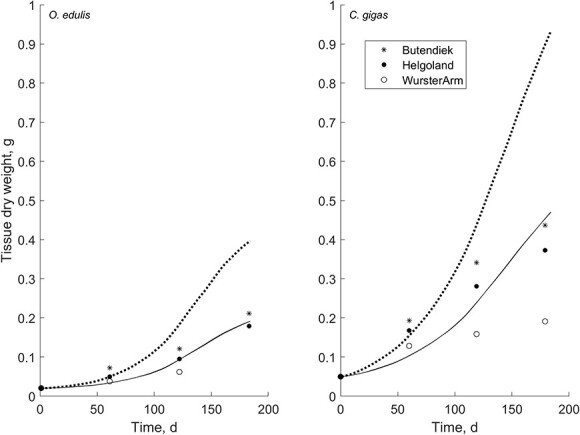
Estimates of aquaculture growth of flat and Pacific oysters in the German Bight (Pogoda *et al.,* 2011) at varying temperature and constant food level. Predictions of growth rates (full line) were similar for the Butendiek site (*O. edulis*, *f* = 0.64; *C. gigas*, *f* = 0.51) and the Helgoland site (*O. edulis*, *f* = 0.63; *C. gigas*, *f* = 0.50). The inshore WursterArm (dashed line) location did not provide good conditions for aquaculture, but high eutrophication levels caused simulations to overestimate food availability and therefore growth (*O. edulis*, *f* = 0.86; *C. gigas*, *f* = 0.77).

Pogoda *et al.* (2011) collected growth data of both flat and Pacific oysters reared at three locations in the German Bight (Fig. 5). The offshore locations, Butendiek and Helgoland, had similar environmental conditions although oysters cultivated at Butendiek grew slightly faster. Growth in the Wursterarm was poor for both oysters, and unsuitable environmental conditions caused the die-off of flat oysters during the experiment.

Simulations of growth at Butendiek and Helgoland were good for both flat ($MRE=0.21$ & $MRE=0.08$) and Pacific oysters ($MRE=0.20$ & $MRE=0.22$). Food conditions resulted in acceptable food availability for flat oysters ($f=0.64$ & $f=0.63$) and a lower food availability for Pacific oysters ($f=0.51$ & $f=0.50$). Estimations of growth at Wursterarm were overestimated for both species, and the witnessed die-off of flat oysters was not represented in the simulations. Eutrophic conditions at Wursterarm were translated in high food availability for flat oysters ($f=0.86$) and Pacific oysters ($f=0.77$).

## Discussion

### General

The flat oyster has been included in the European diet for centuries (Drinkwaard, 1998; Troost, 2010) and is up to now an important cultured species. Both aquaculture and the recent development of flat oyster restoration projects (https://noraeurope.eu/) request the ability to model the metabolic traits of this species. DEB parameters give insight in life history traits. Besides that, the DEB model can serve as an excellent basis to develop temporal or spatial information such as habitat suitability maps (Holbach *et al.,* 2020), estimation of production cycles (Thomas *et al.,* 2011; Palmer *et al.,* 2020), optimal farm configurations (Rosland *et al.,* 2011), site selection (von Thenen *et al.,* 2020), estimation of particle depletion (Taylor *et al.,* 2021) and carrying capacity of production areas (Filgueira *et al.,* 2014).

### Parameter estimation

The DEB theory provides a framework that clearly allows for the simulation of metabolic treats. This parametrization was performed making use of data collected from the North-Atlantic flat oyster population (Launey *et al.,* 2002; Vera *et al.,* 2016) and should be re-validated for applications in the Mediterranean or Black Sea. All metabolic characteristics could be modelled well. However, insufficient information is available in the published literature to calibrate and validate growth and metabolism of D-larvae during brooding. Including an additional dataset of larval growth rate during the brooding period would increase the correctness of parameter values.

### Food availability

Food availability is well studied in shellfish. Energy intake is regulated by filtration, ingestion and assimilation and is quantified by surface-related DEB primary parameters, $\{{F}_m\},\{{P}_{Xm}\}$or $\{{P}_{Am}\}$, a temperature response factor, the structural surface of the individual (${V}^{\frac{2}{3}}$) and the food availability, which is expressed as a functional response related to environmental variables. The food availability is mainly linked to the concentration of food in the water (which is given by the Holling type-ii functional response), but other environmental factors are known to impact feeding fluxes in shellfish. These factors include salinity (Wells, 1961; Robert *et al.,* 1988; Hutchinson and Hawkins, 1992; Guzmán-Agüero *et al.,* 2013; Gosling, 2015), suspended material (Gosling, 2015), current velocity (Walne, 1972; Gosling, 2015), fouling (Hidu *et al.,* 1981; Arakawa, 1990), algal composition (Gerdes, 1983; Picoche *et al.,* 2014), water renewal (Wilson, 1973), cultivation method (Garen *et al.,* 2004; Comeau, 2013), carrying capacity (Ferreira *et al.,* 2008) and sediment composition (Riisgård, 2001). It was deemed out of the scope of this article to quantify functional responses for all the factors, and food availability estimations in the parameterisation process include the influence of all these factors on the feeding mechanisms. It is important to note that erroneous interpretations of growth situations might lead to wrong estimations of food availability levels. For example, environments that support high feeding fluxes (and therefore high food availability) but require high maintenance costs (e.g. Bonamia infections, high wave, or current impacts) will lead to similar observed growth to situations characterized by low feeding fluxes and low maintenance requirements (Filgueira *et al.,* 2020). During the validation step, food availability was linked to chl a only. Situations where estimations matched the observations well therefore imply that chl a concentrations are a suitable proxy for food availability, but estimations that were off indicate that other environmental factors become important.

Food availability at both aquaculture sites in the Danish Limfjorden was different. The harbour area, Lemvig Havn, is used for suspended culture of spat and is located in eutrophic waters. The more open-water station, Gjellergraven, is used for grow-out of oysters in net bags on the bottom and the average chl a concentration is lower. Model-to-data fittings during the last cultivation months at Gjellergraven were off, and other environmental factors (e.g. fouling) were influencing the food availability.

Flat oysters cultured in the German Bight showed excellent growth in offshore conditions with high water renewal and sufficient food. Unsuitable conditions such as those in WursterArm caused total mortality of flat oysters. Pogoda *et al.* (2011) states low salinity during rain, high currents and high sediment loads, which all reduce food intake, as the most probable reason for flat oyster mortalities at this location. The average salinity at Wursterarm was 17.23 ppt (Copernicus Marine: product id: NWSHELF_MULTIYEAR_PHY_004_009). This salinity is suboptimal for flat oysters and inhibits filtration with a factor 0.6 (Hutchinson and Hawkins, 1992). Including the effect of additional environmental drivers on the feeding mechanism will increase model to data fitting and model correctness on a spatial scale. According to the food response calibrated in this work, chl a levels in offshore locations were low for cultivation of *C. gigas.* Cultivation of these cupped oysters in WursterArm did not result into mortalities.

### Temperature response

The temperature response for *O. edulis* was estimated based on growth experiments in the laboratory at different temperatures and food availability. Growth experiments show reduced growth at 30°C. Experiments performed by Eymann *et al.* (2020) showed a reduced filtration rate at temperatures higher than 26°C and the onset of cardiac dysfunctions and anaerobiosis in the gills. The study describes the optimal temperature for *O. edulis* being between 18°C and 23°C for specimen obtained in Northern Spain. Nevertheless, no growth inhibition at 30°C was observed during larval growth experiments in the laboratory (Robert *et al.,* 2017) and filtration rate, which drives growth, did not decrease at 30°C in the experiments of Haure *et al.* (1998). Both publications did not mention the origin of the oysters. At the lower temperature range, similar inconsistencies have been witnessed. Flat oyster growth data collected by Wilson (1987) showed no observable growth in Birterbuy Bay, Galway, Ireland, during the winter season (min. temperature, 8°C), while the experiments performed by Kamermans and Saurel (2022) clearly show an increase in oyster tissue at temperatures of 8°C. Plasticity of the thermal tolerance has been described in oysters (Bible *et al.,* 2020) and we therefore advice recalibration of T_H_ and T_L_ whenever modelling oyster growth at extreme temperatures. In addition, the temperature response could be improved at these extremes by decoupling of the Arrhenius relationship for ingestion rate and for respiration rate as done by Bourlès *et al.* (2009). Child *et al.* (1998) published insights in the survival and growth of oysters exposed to low temperatures and found that exposure of *C. gigas* to temperatures below 3°C causes mortality. During the laboratory experiments performed in this study, which lasted slightly more than a month, no moralities of *C. gigas* was observed. However, growth at 3°C was very limited for both *C. gigas* and *O. edulis*.

The metabolic temperature response of both oysters is very similar, and although flat oysters have historically been present in Sweden and Norway, sightings of *C. gigas* in Northern Europe were limited. Threshold temperatures such as minimum temperature for larval settlement and larval survival are seen as important limitations for the habitat expansion of *C. gigas* towards northern countries. Simulations performed by the Norwegian Institute for Water Research (Report No. 7016-2016) showed that temperatures in the 1990s were too cold for successful larval development and survival. Recent increases in summer seawater temperatures facilitated successful spatfall in Northern Europe (Bodvin *et al.,* 2010; Laugen *et al.,* 2016; Shelmerdine, 2017; Bergström *et al.,* 2021). Warming winter temperatures, on the other hand, will reduce winter mass mortality of *O. edulis* populations (Cole, 1940). Higher water temperatures also has positive effects on both the reproductive output and the physical environment where larvae and settling juveniles are living (Spärck, 1925; Orton, 1927; Spärck, 1949; Hart *et al.,* 2011). Several successive warm summers have already induced larger spat fall of flat oysters in the Limfjorden (Nielsen and Petersen, 2019).

### Life history traits based on DEB

At the inter-species level, the body size scaling relationships implied by DEB theory provided us with a simple but successful way to compare life history traits among the different species. Both the European flat oyster and the Pacific oyster follow a typical bivalve life cycle. Flat oyster eggs are large compared with Pacific oyster eggs and contain up to 10 times more energy (3.7 10^−7^ J). Investments in energy-rich eggs increase viability of the embryo’s but reduce offspring clutch size. Fertilization of the eggs and embryo development takes place inside the mantle cavity of the female flat oyster (Chaparro *et al.,* 1993; Bayne, 2017), which classifies them as an ovo-viviparous species. Brooding is represented in DEB through a high age at birth (5.16 days past fertilization), which implies that feeding during the first days of life are limited. For Pacific oysters, fertilization takes place in the water column and feeding starts early (1.12 days past fertilization). Flat oyster larvae are released at a size of 200 μm according to the DEB parameters, which is slightly higher than the length of 180 μm mentioned in the literature (Labarta *et al.,* 1999; Araya *et al.,* 2012; Robert *et al.,* 2017). Larval readiness to be released is translated into an additional DEB maturity parameter, ‘Maturity level at release (${E}_H^r=0.0006\ J$)’, which does not imply altered metabolism, but merely serves as an important life event. Flat oyster larvae settle at a size of around 380 μm according to DEB, or 270–300 μm according to the literature (Labarta *et al.,* 1999; Araya *et al.,* 2012; Robert *et al.,* 2017). During metamorphosis of both oyster species, which coincides with settlement, the velum disappears and is replaced by the formation of gills and filtration is reduced. Reserves and structure are used to cope with the decrease in energy assimilation during metamorphosis (Labarta *et al.,* 1999; Robert *et al.,* 2017). Oyster larvae follow linear growth during their pelagic phase, until they are ready to settle (${E}_H^s=0.004\ J$ for flat oysters and ${E}_H^s=0.002\ J$ for Pacific oysters). Flat oysters spend double the amount of energy on maturation during their pelagic phase, compared with Pacific oyster larvae, implying strong investments in the development (including the immune system and metamorphosis). After settlement, growth rate increases exponentially (Labarta *et al.,* 1999), which is represented by the acceleration factor in the ‘asj’ DEB model (Kooijman, 2014). For *C. gigas* (${s}_M=4.8$), acceleration goes slower compared with *O. edulis* (${s}_M=5.9)$. Pacific oysters also reach puberty earlier, which enables them to reproduce at a younger age. Adult flat oysters can grow up to 17 cm with total weights of almost 500 g and reach a lifetime of up to 30 years (Saurel and Richardson, 2003).

Flat oysters are sequential hermaphroditic and change sex during the year. Both sexes are generally present in the population throughout the year and sex changes depend on energy availability (male to female) and spawning events (female to male) (Orton, 1924; Cole, 1941). Both male and female reproductive output is modelled in the DEB model for *O. edulis* by a loss of reproduction buffer by ${\kappa}_r=0.45$, assuming an even energy allocation to sperm and eggs. Although cupped oysters are sequential hermaphroditic (Broquard *et al.,* 2020), sex determination has not been included in the published DEB models.

### The parameter values *C. gigas* vs *O. edulis*

Two parameter sets have been used for estimating metabolic traits of *C. gigas* (Table 2). The parameters published in the AmP database have been estimated based on a comprehensive dataset, which is mainly focused on the early life stages (Emmery *et al.,* 2011; Emmery and Kooijman, 2015). A second set of DEB parameters have been published by Pouvreau *et al.* (2006) and is intensively used for the estimation of aquaculture purposes and suitability mapping (Bourlès *et al.,* 2009; Grangeré *et al.,* 2009; Thomas *et al.,* 2016; Palmer *et al.,* 2020). These parameters are standard DEB parameters and do not cover metabolic acceleration during metamorphosis. The parameter set of flat oysters and both parameter sets of Pacific oysters are compiled in Table 2, to allow for easy comparison. An extensive read-through of the description of each DEB parameter can be found in Kooijman (2010).

**Table 2:** DEB parameters for flat and Pacific cupped oysters

Symbol	Units	*O. edulis*	*C. gigas*(Add my Pet)	*C. gigas*(Pouvreau *et al.,* 2006)	Description
		*‘Asj’*	*‘Asj’*	*‘std’*	
${T}_{ref}$	$K$	293.1	293.1	293.1	Reference temperature
${T}_A$	$K$	5000	5800	5800	Arrhenius temperature
${T}_L$	$K$	286	281	281	Lower boundary tolerance range
${T_A}_L$	$K$	23 000	75 000	75 000	Arrhenius temp. low boundary
${T}_H$	$K$	303	300	300	Upper boundary tolerance range
${T}_{AH}$	$K$	55 610	30 000	30 000	Arrhenius temp. upper boundary
$\boldsymbol{z}$	$-$	**1.06 (6.03)**	**1.18 (5.62)**	**4.29**	**Zoom factor**
${F}_m$	$l\ {d}^{-1}\ c{m}^2$	13.86	30.4		{F m}, max spec searching rate
$\{{\boldsymbol{p}}_{\boldsymbol{Am}}\}$	$\boldsymbol{J}\ {\boldsymbol{d}}^{-\mathbf{1}}\ \boldsymbol{c}{\boldsymbol{m}}^{\mathbf{2}}$	**91**	**372**	**315**	**Max spec assimilation rate**
${\kappa}_X$	$-$	0.8	0.3259	0.75	Digestion efficiency of food to reserve
${\kappa}_P$	$-$	0.1	0.2279	0.05	Faecation efficiency of food to faeces
$\boldsymbol{v}$	$\boldsymbol{cm}\ {\boldsymbol{d}}^{-\mathbf{1}}$	**0.020**	**0.0054**	**0.18**	**Energy conductance**
$\boldsymbol{\kappa}$	$-$	**0.92**	**0.26**	**0.45**	**Allocation fraction to soma**
${\kappa}_R$	$-$	0.45	0.95	0.75	Reproduction efficiency
$[{\boldsymbol{p}}_{\boldsymbol{M}}]$	$\boldsymbol{J}\ {\boldsymbol{d}}^{-\mathbf{1}}\boldsymbol{c}{\boldsymbol{m}}^{\mathbf{3}}$	**15.68**	**17.35**	**44**	**[p M], vol-spec somatic maintenance**
$\{{p}_T\}$	$J\ {d}^{-1}c{m}^2$	0	0	0	{p T}, surf-spec somatic maintenance
${k}_J$	$1\ {d}^{-1}$	0.002	0.002	0.002	Maturity maintenance rate coefficient
${E}_G$	$J\ c{m}^{-3}$	2374	2374	3900	[E G], spec cost for structure
${E}_{H_b}$	$J$	5.002e-4	1.78e-4		Maturity at birth
${E}_{H_r}$	$J$	5.621e-4			Maturity at release
${E}_{H_s}$	$J$	4.025e-3	1.858e-4		Maturity at settlement
${E_H}_j$	$J$	8.637e-1	2.076e-2		Maturity at metamorphosis
${{\boldsymbol{E}}_{\boldsymbol{H}}}_{\boldsymbol{p}}$	$\boldsymbol{J}$	**32**	**1.778**		**Maturity at puberty**
${h}_a$	${d}^{-2}$	6.224e-10	1.03e-9		Weibull ageing acceleration
${s}_g$	$-$	0.0001	0.0001		Gompertz stress coefficient
${\delta}_{M_b}$	$-$	0.6992	0.87		Shape coefficient before metamorphosis
${\delta}_M$	$-$	0.397	0.20	0.18	Shape coefficient after metamorphosis

The **zoom factor**, $\boldsymbol{z}$, is defined as $z={L}_m/{L}_{ref}$ with ${L}_m=\kappa .{p}_{Am}/{p}_M$ and ${L}_{ref}=1 cm$. The zoom factor approaches the ultimate wet weight (not considering the weight of the reproduction buffer) to the power 1/3. Organisms with high zoom factors therefore reach a larger ultimate size. As seen in Table 2, the estimated zoom factor for both oyster species, and therefore also their ultimate size is comparable. Feeding characteristics in DEB theory are represented by the primary parameters, **maximum specific searching rate**, $\{{\boldsymbol{F}}_{\boldsymbol{m}}\}$, (which relates to the clearance rate) and the **digestion efficiency**, ${\kappa}_X$, (which relates to the assimilation efficiency). The **maximum volume specific ingestion rate**, $\{{p}_{Xm}\ \}$, and the **maximum volume specific assimilation rate**, $\{{p}_{Am}\}$, are compound parameter that drive ingestion and assimilation. Feeding traits of both oyster species were evaluated and compared by Nielsen *et al.,* (2017) and very different clearance rates were found for both species (6.5–12.6 L h^−1^ g^−1^ for flat oysters, and 8.5–27.1 L h^−1^ g^−1^ for Pacific oysters). Retention efficiencies (between 80% and 100%) were reported to be similar for both species (Nielsen *et al.,* 2017), which implies that Pacific oyster ingestion rates are higher than those of flat oysters because clearance rates are higher. This largely coincides with the results of this study. Assimilation efficiency (corresponding to digestion efficiency in DEB) was measured to be between 0.5 and 0.8 (Hutchinson and Hawkins, 1992) for flat oysters depending on temperature and salinity and 72.5% for Pacific oysters (Gerdes, 1983). Digestion efficiencies of Pacific oysters seem to be underestimated in the DEB parameter set of Emmery and Kooijman (2015). Flat oysters have higher **energy conductance**, $\dot{\boldsymbol{v}}$, which controls the reserve mobilization rate, compared with the cupped oyster. They therefore also have a smaller maximum reserve capacity ($[{E}_m]=\{{p}_{Am}\}/\dot{v}$), a smaller reserve residence time (${t}_E=L/\dot{v}$) and a lower contribution of the reserve to the total dry weight. This all indicates a profound resistance of *C. gigas* to prolonged starvation compared with *O. edulis*. Low energy conductance values in intertidal species have been published and discussed before in the intertidal rock oysters (Chowdhury *et al.,* 2018), the Antarctic limpet (Guillaumot *et al.,* 2020) and intertidal mussels (Tagliarolo *et al.,* 2016).

The **volume specific maintenance rate** is lower for *O. edulis*, which indicates that it spends less energy on maintenance, and therefore can survive at lower constant food levels: if assimilated energy does not drop below $[{\dot{p}}_M].{L}^3$ the organism does not experience starvation. Intertidal organisms are generally characterized by a higher volume specific maintenance cost ($J\ {cm}^{-3}$) implying that they have to spend more energy maintaining homeostatic conditions.

Flat oysters grow slow and reach a smaller ultimate size (compared with *C. gigas*). They can survive in habitats with low food availability, which is also represented by the lower **half saturation constant**. But when food conditions are insufficient to counter maintenance costs, they will rapidly deplete reserves. *Ostrea edulis* populations are generally fully submerged and experience a relatively constant supply of food. Although temporal variability exists in food quality and food availability, they do not require extensive energy storage to withstand prolonged periods of starvation.

*Crassostrea gigas* on the other hand, is an intertidal species. The variable nature of intertidal environments require sufficient energy storage to survive. Intertidal habitats such as rocky shores, harbours and estuaries are dynamic systems and filtration might be reduced by low salinity during prolonged rains, bad water quality or high sediment loads. Air exposure makes food intake impossible, and sun exposure increases body temperature and therefore maintenance costs. Whenever conditions are optimal, *C. gigas* will grow very fast, and prolonged optimal conditions will result in oysters with large sizes.

Besides growth, reproductive strategy significantly differs between both species. $\kappa$-values below 0.4 are rare in the animal kingdom. The **allocation to soma** is extremely different between flat and Pacific oyster ($\kappa =0.92$ for flat oysters and $\kappa$ = 0.26–0.45 for Pacific oyster). These extremely low $\kappa$-values witnessed in the DEB parameters of Pacific oysters indicates that partitioning of mobilized energy favours reproduction above growth. Large energy investments in reproduction in combination with low energy content of eggs (large clutch size) are typical characteristics of early colonizers. These characteristics counter predation losses, enlarge the suitable niche through the release of a genetically diverse pool of offspring and increases the speed of evolution, which is beneficial in changing environments.

**Figure 6:**
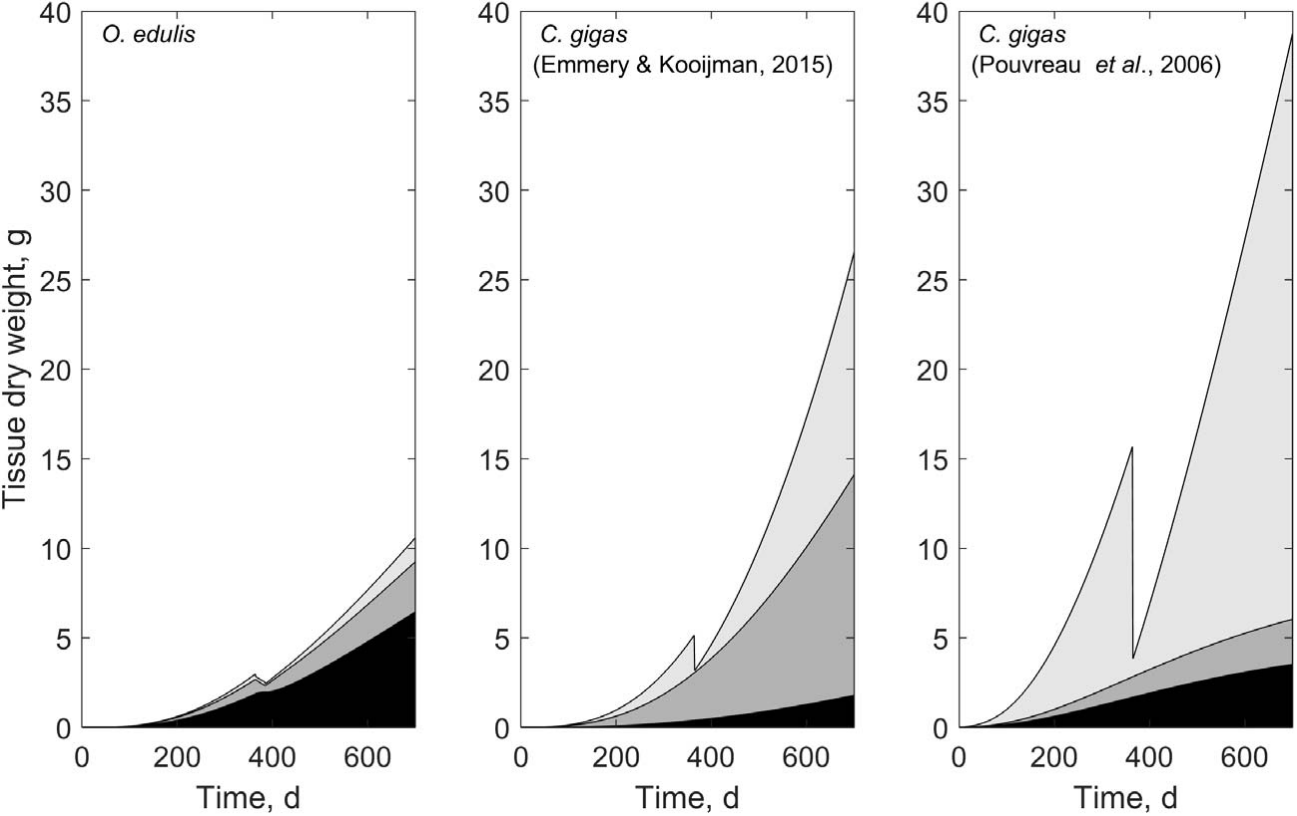
Simulations of tissue dry weight subdivided into structure (black), reserve (grey) and reproduction buffer (light grey) during the first 2 years of life, at optimal food availability and temperature.

Flat oysters on the other hand, allocate less energy into the reproductive products. Brooding species protect their larvae during their most vulnerable phases and can, therefore, reduce reproductive output and energy investment in reproductive products. The *Ostrea chilensis* is an extreme example, since it has a brooding period that lasts until the larvae are ready to settle, and therefore only allocates very limited energy to reproduction ($\kappa =0.996$) (Stechele, 2020). Nevertheless, brooding comes at a cost, and it imposes starvation stress to the brooding animals that reduce filtration. The overall fitness of the brooding animals reduces and mortality after spawning is often witnessed in oysters (Chaparro *et al.,* 2006; Mardones-Toledo *et al.,* 2015). Unfavourable conditions during the brooding period could result in failed recruitment and low fitness of females (Fig. 6).

### Competition and global change

Several studies have shown that *C. gigas* and *O. edulis* co-occur is mixed intertidal reefs (Bergström *et al.,* 2021; Zwerschke *et al.,* 2018) and they, at least partially, occupy similar habitats and feeding niches (Nielsen *et al.,* 2017). In habitats where both species occur, feeding competition will occur, but quantification of the competitive pressure they will impose on each other will depend on population size. While flat oysters are less susceptible to low food availability and the half saturation food constant of flat oysters is low, Pacific oysters graze more intensely.

Climate change is known to impose changes on coastal areas, such as increased air temperature, increased storm frequency, elevated seawater temperature, a decreasing seawater pH and ice coverage and changes in upwelling, river runoff and algae blooms (Harley *et al*., 2006). In general, raising temperatures will boost the reproductive output of both *C. giga*s and *O. edulis* and will increase the growth potential of both species. Warming seawater and air temperature in combination with decreasing alkalinity will increase the maintenance costs of intertidal populations. The impact of these factors and synergistic effects on the performance of the *Ostrea* and the *Crassostrea* genus have recently been a focus of study and concluded that larvae from brooding species were more tolerant to ocean acidification (in combination with other pressures) compared with broadcaster species (Gazeau *et al.,* 2013; Ko *et al.,* 2014; Cole *et al.,* 2016; Gray *et al.,* 2019; Navarro *et al*., 2020).

Alterations in storm frequency, upwelling, river runoff and algae blooms relate to altered food availability dynamics for oysters mainly located in inshore, nearshore or shallow habitats. The DEB parameters of *O. edulis* show that the species is more susceptible to starvation, in comparison to *C. gigas*, and *O. edulis* individuals are especially vulnerable to energy deprivation during the metamorphosis and the brooding period. *Crassostrea gigas* therefore has an advantage over flat oysters in highly dynamic coastal systems, and climate change might invoke additional pressure to depleted coastal *O. edulis* stocks.

In the light of these results, further efforts should be made on the protection of the remaining offshore populations or restoration efforts to be allocated to locations specified by stable and suitable environmental conditions for *O. edulis*.

## Funding

This study was supported by an Australian Research Council Discovery grant (DP190102152 to C.E.F. and R.C.), the Research Foundation–Flanders (FWO) through an SB PhD fellowship (project number 1S84619N to B.S.), the H2020 FORCOAST project (grant agreement number 870465 to M.M.) and the FutureMARES project through the European Union’s Horizon 2020 research and innovation program (grant agreement no 869300 to J.W. and M.M.). This project has received funding from the European Union's Horizon 2020 Research and Innovation Programme under Grant Agreement no 862915.

## Data Availability Statement

The scripts and data underlying this article will be shared on reasonable request to the corresponding author.

## Supplementary Material

suppl_coac034
