# Supplementary material for: Comparing life history traits and tolerance to changing environments of two oyster species (Ostrea edulis and Crassostrea gigas) through Dynamic Energy Budget theory
Source: Conserv Physiol. 2022 Jul 8;10(1):coac034. doi: 10.1093/conphys/coac034 (PMC9271014; doi:10.1093/conphys/coac034)
Supplement: suppl_coac034 [file suppl_coac034.zip › DEB_Formulas.docx]

# Addendum I

1. **DEB model general**

According to the DEB model, 4 state variables define the metabolism of an individual in it’s environment. Both energy reserve and scaled reserve density are used to define the fitness of the animal, while both strutural volume and structural length are used to define the size of the animal.

| State variable | Unit | Name |
| --- | --- | --- |
| $V$ | $cm^{3}$ | Structural volume |
| $E$ | $J$ | Energy reserve |
| $E_{R}$ | $J$ | Reproduction buffer |
| $E_{H}$ | $J$ | Maturity level |
|  |  |  |
| $L=V^{\frac{1}{3}}$ | $cm$ | Structural length |
| $\left[ E \right]=\frac{E}{V}$ | $J cm^{-3}$ | Reserve density |
| $e=\left( \frac{E}{V E_{m}} \right)$ | - | Scaled reserve density (fitness, between 0 and 1) |

State variables can be converted to measurable characteristcs of the organism such as length (cm) and weights (g) amongst many other.

**Lenght**

DEB theory links the physical length *Lw* (*cm*) to state variable structural volume, $V (cm^{3}$) (eq. 1) using *δ_m_* which is the dimensionless shape correction factor. The shape correction factor is only constant for an organisms that stay isomorphic over the course of its life (Kooijman, 2000).

$L_{w}=\frac{V^{\frac{1}{3}}}{\delta_{M}}$ (1)

**Weight**

DEB theory describes the body weight of an organism as the sum of the weight of the structure and the energy reserves $(E+E_{R})$. Dry weights (eq. 2) are converted to wet weights (eq. 3) using conversion factors for structure and volume.

$Wd=d_{v} V+\left( E+E_{R} \right)\frac{w_{Ew}}{\mu_{E}}$ (2)

$Ww=V +\left( E+E_{R} \right) \frac{w_{Ew}}{\mu_{E} . d_{E}}$ (3)

Used auxiliary parameters for these conversions are:

| Parameter | Unit | Value | Name |
| --- | --- | --- | --- |
| $d_{vw}$ | $g cm^{-3}$ | 1 | Density of wet structure |
| $d_{E}$ | - | 0.09 | Wet to dry conversion factor for energy |
| $d_{V}$ | - | 0.09 | Wet to dry conversion factor for structure |
| $w_{Ew}$ | $g mol^{-1}$ | 23.9 | Molar weight of the reserve |
| $\mu_{E}$ | $J mol^{-1}$ | 550 000 | Chemical potential of the reserve |

1. **DEB coupling with the environment**

Several variables are known to influence the metabolism. In this study, the influence of food availability and temperature is included. Forcings for these drivers are sea surface temperature, chlorophyll a and suspended particulate matter (SPM) are considered as forcings.

**Temperature**

The temperature response is represented throught the ’temperature correction factor’ which is calculated according to the Arrhenius equation (eq. 4).

$c_{T}=e^{(\frac{T_{A}}{T_{ref}}-\frac{T\_A}{T})}$ (4)

**Food availability**

Food avilaiblity, $f$ relates to the concentration of the food in the water according to equation 5. In case filtration inhibition occurs through high concentrations of SPM, $f$ is calculated according to equation 6.

$f=\frac{X}{X+K_{X}}$ (5)

$f=\frac{X}{K_{X} \left( 1+\frac{Y}{K_{Y}} \right)}$ (6)

| Variable | Unit | Name |
| --- | --- | --- |
| $T$ | $K$ | Sea surface temperature |
| $X$ | $\mu g L^{-1}$ | Chlorophyll a concentration |
| $Y$ | $mg L^{-1}$ | Concentration SPM |

1. **DEB model dynamics**

From the primary parameters, compound parameters are calculated:

| Parameter | Unit | formulation | name |
| --- | --- | --- | --- |
| $\left\{ p_{Am} \right\}$ | $J d^{-1}cm^{-3}$ | $\left\{ p_{Am} \right\}=z \frac{p_{M}}{\kappa}$ | Surface specific assimilation |
| $\left[ E_{m} \right]$ | $J cm^{-3}$ | $\left[ E_{m} \right]= \frac{p_{Am}}{v}$ | Max. reserve density |
| $g$ | - | $g=\frac{E_{G}}{\left( \kappa\left[ E_{m} \right] \right)}$ | Energy investment ratio |
| $k_{M}$ | $d^{-1}$ | $k_{M}=\frac{p_{M}}{\left[ E_{G} \right]}$ | Somatic maintenance rate coefficient |
| $L_{m}$ | $cm$ | $L_{m}=\frac{v}{k_{M} g}$ | Maximum structural length |
| $L_{b}$ | $cm$ |  | Strucural length at birth |
| $L_{j}$ | $cm$ |  | Structural length at end metamorphosis |
| $k_{J}$ |  |  |  |

Primary parameters and compound parameters are adapted to life stage $(s_{M})$ and temperature:

$\dot{v_{T}}=\dot{v} c_{T} s_{M}$

$k_{JT}=k_{J} c_{T}$

$L_{m_{j}}=L_{M} s_{M}$

With

$s_{M}=\min\left( \frac{L_{j}}{L_{b}},\frac{L}{L_{b}} \right)$ (7)

**Changes in state variables over time**

The values of state variables at certain times were computed by solving differential equations.

Changes in **structural length,** $\frac{dL}{dt}$ are related to the specific volumetric growth rate $r$ (*d*^−1^);

$\dot{r}= \frac{\dot{v_{T}} (\frac{e}{L}-\frac{1}{L_{mj}})}{(e+g)}$ (8)

$\frac{dL}{dt}=L \frac{\dot{r}}{3}$ (9)

The **reserve density** at steady state is given by $\left[ E \right]= f \left\{ p_{Am} \right\} / \dot{v}$. The maximum reserve density $[Em]$ occurs when food availability is optimal $(f = 1).$ Therefore, the changes in scaled reserve density $e$ is expressed as:

$\frac{de}{dt}=\left( f-e \right) \frac{\dot{v}}{L}$ (10)

Changes in **reproduction buffer (**$\boldsymbol{E}_{\boldsymbol{R}}$**)** or **maturity level (**$\boldsymbol{E}_{\boldsymbol{H}}$**)** are described by:
$\frac{dE_{H}}{dt}=\left( 1-\kappa\right) \dot{p}_{c}-E_{H} k_{J} \mathrm{and} \frac{dE_{R}}{dt}=0 \mathrm{when} E_{H}=<E_{Hp}$ (11)

$\frac{dE_{H}}{dt}= 0 \mathrm{and} \frac{dE_{R}}{dt}=\left( 1-\kappa\right) \dot{p}_{c}-E_{Hp} k_{J} \mathrm{when} E_{H}>E_{Hp}$ (12)

with $\dot{p}_{C},$ the energy mobilization from reserve:
$\dot{p}_{c}=\left( \frac{\dot{v_{T}}}{L}-\dot{r} \right) e \left[ E_{m} \right] L^{3}$ (13)

**Fluxes**

Energy fluxes $(J d^{-1})$through the animal are calculated according to:

$\dot{CR}= \left\{ F_{m} \right\} L^{2} f c_{T} s_{M}$ (14) Clearance rate

$\dot{p}_{X}= \left\{ p_{Am} \right\} \kappa_{X} L^{2} f c_{T} s_{M}$ (15) Ingestion rate

$\dot{p}_{A}=\left\{ p_{Am} \right\} f L^{2} c_{T} s_{M}$ (16) Assimilation rate

$\dot{p}_{c}=\left[ E_{m} \right] \left( \dot{vT} L^{2}+k_{MT} L^{3} \right) \frac{\left( e . g \right)}{(e+g)}$ (17) Mobilization rate

$\dot{p}_{S}= p_{MT} V$ (18) Somatic maintenance rate

$\dot{p}_{J}= E_{H} k_{JT}$ (19) Maturity maintenance rate

$\dot{p}_{G}= \kappa\dot{p}_{c}- \dot{p}_{s}$ (20) Growth rate

$\dot{p}_{R}=\left( 1-\kappa\right) \dot{p}_{c}- \dot{p}_{J}$ (21) Reproduction rate

$\dot{p}_{D}=\dot{p}_{S}+\dot{p}_{J}+\left( 1- \kappa_{R} \right) \dot{p}_{R}$ (22) Dissipation rate

**Oxygen consumption, carbon dioxide excretion and ammonia excretion**

Translating energy fluxes to organic fluxes from food $\dot{(J}_{X})$ to structure $\dot{(J}_{V})$, to reserves $\dot{(J}_{E})$ and to faeces $\dot{(J}_{R})$, expressed in $cmol d^{-1},$ is done by multiplying the energy fluxes with mass-energy couplers $\left( cmol J^{-1} \right).$ Conversions are done using the chemical potentials of reserve and yields of food to energy $(y_{X_{E}}, \frac{mol}{mol})$ and faeces on reserve $\left( {y_{P}}_{E}, \frac{mol}{mol} \right)$ and organic fluxes only depend on the assimilation flux, growth flux and dissipation flux.

$\dot{J}_{X}= \dot{p}_{A} (-\eta_{XA})= \dot{p}_{A} (-\frac{y_{X_{E}}}{\bar{\mu}_{E}})$ (23)

$\dot{J}_{V}= \dot{p}_{G} (-\eta_{VG})= \dot{p}_{G} (-\frac{y_{V_{E}}}{\bar{\mu}_{E}})$ (24)

$\dot{J}_{E}+\dot{J}_{ER} =\frac{\dot{p}_{A}}{\bar{\mu}_{E}}- \frac{\dot{p}_{D}}{\bar{\mu}_{E}} -\frac{\dot{p}_{G}}{\bar{\mu}_{E}}$ (25)

$\dot{J}_{P}= \dot{p}_{A} \eta_{PA}= \dot{p}_{G} \left( -\frac{y_{X_{P}}}{\bar{\mu}_{E}} \right)$ (26)

Conversions between energy fluxes and organic fluxes can also be performed using a matrix multiplication

$\dot{J}_{O}=\left[ \begin{matrix} \dot{J}_{X} \\ \dot{J}_{V} \\ \dot{J}_{E}+\dot{J}_{ER} \\ \dot{J}_{P} \end{matrix} \right]= \left[ \begin{matrix} \dot{p}_{A} \\ \dot{p}_{D} \\ \dot{p}_{G} \end{matrix} \right] \left[ \begin{matrix} -\eta_{XA} & 0 & 0 \\ 0 & 0 & \eta_{VG} \\ {\bar{\mu}_{E}}^{-1} & {-\bar{\mu}_{E}}^{-1} & -{\bar{\mu}_{E}}^{-1} \\ \eta_{PA} & 0 & 0 \end{matrix} \right]$ (23 – 26)a

$\dot{J}_{O}=\left[ \begin{matrix} \dot{J}_{X} \\ \dot{J}_{V} \\ \dot{J}_{E}+\dot{J}_{ER} \\ \dot{J}_{P} \end{matrix} \right]= \left[ \begin{matrix} \dot{p}_{A} \\ \dot{p}_{D} \\ \dot{p}_{G} \end{matrix} \right] \left[ \begin{matrix} -2.38{10}^{-6} & 0 & 0 \\ 0 & 0 & 1.59{10}^{-6} \\ 1.82 {10}^{-6} & -1.82 {10}^{-6} & -1.82{10}^{-6} \\ 2.66{10}^{-7} & 0 & 0 \end{matrix} \right]$ (23 – 26)b

**Organic fluxes** can be converted to elementary composition using: 1:1.8:0.5:0.15 ratio of carbon to hydrogen, oxygen and nitrogen in organic compounds ($n_{O}$). Elementary composition of food, structure, energy storage and faeces are calculated by multiplying $\dot{J}_{O}$ with $n_{O}$.

$\begin{matrix} X & V & E & P \end{matrix}$

$n_{0}=\left[ \begin{matrix} 1 & 1 & 1 & 1 \\ 1.8 & 1.8 & 1.8 & 1.8 \\ 0.5 & 0.5 & 0.5 & 0.5 \\ 0.15 & 0.15 & 0.15 & 0.15 \end{matrix} \right] \begin{matrix} C \\ H \\ O \\ N \end{matrix}$

Fluxes of the **mineral fractions** (CO_2_, H_2_O, O_2_ and NH_3_) are called mineral fluxes $(\dot{J}_{M})$ and are expressed in mol/d. The elementary composition of the mineral fractions is stored in $m_{O}$.

$\begin{matrix} C & H & O & N \end{matrix}$

$\dot{J}_{M}=\left( \begin{matrix} \dot{J}_{C} \\ \dot{J}_{H} \\ \dot{J}_{O} \\ \dot{J}_{N} \end{matrix} \right)$ and $m_{0}=\left[ \begin{matrix} 1 & 0 & 0 & 0 \\ 0 & 2 & 0 & 3 \\ 2 & 1 & 2 & 0 \\ 0 & 0 & 0 & 1 \end{matrix} \right] \begin{matrix} C/C \\ H/C \\ O/C \\ N/C \end{matrix}$

Due to the conservation of mass, the following mass balance can be made

$0=n_{M} \dot{J}_{M} +n_{O} \dot{J}_{o}$ (27)

Or

$\dot{J}_{M}= {-(n_{M}}^{-1} n_{O} \dot{J}_{O})$ (28)

The first element of $\dot{J}_{M}$ then represents the excretion of CO_2_, the second element the excretion of H which is barely used, the third element contains the consumption of O (from O_2_) and the last element the excretion of NH_3_, all expressed in mol/day.
